# A rare case of anti-LGI1 limbic encephalitis with concomitant positive NMDAR antibodies

**DOI:** 10.1186/s12883-020-01918-7

**Published:** 2020-09-07

**Authors:** Tuo Ji, Zhi Huang, Yajun Lian, Chengze Wang, Qiaoman Zhang, Jinghong Li

**Affiliations:** Department of neurology, First Affiliated Hospital of Zhengzhou University, Zhengzhou City, 450052 Henan Province China

**Keywords:** Anti-leucine-rich glioma-inactivated 1, Limbic encephalitis, N-methyl-D-aspartate receptor, Antibody

## Abstract

**Background:**

N-methyl-D-aspartate receptor (NMDAR) and leucine-rich glioma-inactivated 1 (LGI1) antibodies define the most prevalently recognized autoimmune encephalitis syndromes, while the simultaneous occurrence of both conditions has hardly been published before.

**Case presentation:**

We report the case of a 67-year-old patient who presented with generalized tonic–clonic seizures (GTCS) followed by behavioral changes, psychosis, sleep disorders, decreased consciousness, and faciobrachial dystonic seizures. Ancillary findings included serum hyponatremia and imaging evidence of high-intensity lesions within bilateral medial temporal lobes on T2-weighted fluid attenuation inversion recovery. Both LGI1 and NMDAR antibodies were positive in serum and cerebral spinal fluid using transfected cell based assays. Despite prominent clinical features of anti-LGI1 limbic encephalitis (LGI1-LE), the patient also exhibited overlapping symptoms of anti-NMDAR encephalitis, like early-onset GTCS, which might be related to the concomitant positive NMDAR antibodies.

**Conclusions:**

We report a rare case of LGI1-LE with overlapping symptoms and simultaneous positive NMDAR antibodies. It is necessary to evaluate the presence of NMDAR antibodies in certain LGI1-LE patients with unusual symptoms. However, caution should be exercised in interpreting the observation, given the fact of a single-case study.

## Background

Anti-leucine-rich glioma-inactivated 1 limbic encephalitis (LGI1-LE) is an auto-antibody mediated disorder characterized by an acute to sub-acute onset of confusion and cognitive impairment, facio-brachial dystonic seizures (FBDS) and psychiatric disturbances [1, 2]. Anti-N-methyl-D-aspartate receptor (NMDAR) encephalitis is a severe autoimmune nervous system disease, the major symptoms of which include abnormal behavior or cognitive dysfunction, speech dysfunction, seizures, movement disorders or dyskinesia or abnormal posture, decreased consciousness, and autonomic dysfunction or central hypoventilation [3]. NMDAR and LGI1 antibodies define the most prevalently recognized autoimmune encephalitis (AE) syndromes, while the simultaneous occurrence of both conditions has hardly been published before [4]. We herein describe the case of a 67-year-old man with LGI1-LE presenting overlapping symptoms and simultaneous positive NMDAR antibodies. The aim of this report is to add knowledge on the possible clinical spectrum of anti-LGI1 and anti-NMDAR overlapping syndromes.

## Case presentation

A 67-year-old Chinese male was admitted to the hospital in his hometown with two episodes of witnessed generalized tonic–clonic seizures (GTCS) with no aura in November 2018. Thereafter, he developed hallucinations, delusions and short-term memory loss, although he did not complain of headache, nausea, or fever. Shortly after admission, he became increasingly confused, and showed impulsiveness and irritability, and using foul language. Sleep wake pattern was altered with increased drowsiness during the day and insomnia at night. Several days later, he developed short, jerky, unilateral, involuntary movements predominantly of the left, but occasionally also of the right arm and face, suggestive of facio-brachial dystonic seizure (FBDS). The episodes lasted about a second, occurred several times per day, and were occasionally associated with involuntary vocalizations. The first magnetic resonance imaging (MRI) scan of the brain was unremarkable (imaging not available). His symptoms partially improved following treatment with oral carbamazepine and mannitol, after which he was discharged home. Ten days after discharge, he suffered another episode of GTCS, and was transferred to our hospital to check for possible etiologies in January 2019. Following admission, he was partially oriented, disinhibited and with a depressed mood. He also developed intermittent visual hallucinations, paranoia and involuntary facio-brachial movements. The disease rapidly progressed, and the disturbance of consciousness changed from lethargy to coma.

Past medical history was unremarkable except hypertension for 1 year. He denied history of previous herpes simplex virus encephalitis (HSE). He did not smoke, drink alcohol or use any illicit drugs. There was no family history of genetic diseases and autoimmune diseases. On admission, a neurologic examination revealed drowsiness and decreased responsiveness. He was partially oriented to time and place. Cranial nerve examination remained intact. Motor exam revealed normal muscle strength. Finger-to-nose and heel-to-shin testing were normal. Bilateral Babinski’s signs were present.

**Fig. 1:**
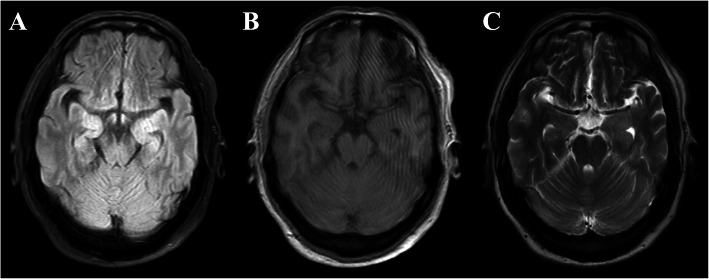
Magnetic resonance imaging of the patient. T2-weighted fluid-attenuated inversion recovery (FLAIR) showed slightly elevated signals within bilateral medial temporal lobes (**a**), whereas no abnormalities were seen on the T1 or T2 scans (**b**-**c**)

On initial evaluation at our facility, a brain MRI revealed abnormal hyperintense signals within bilateral mesial temporal lobes on fluid attenuation inversion recovery (FLAIR) (Fig. 1). No abnormalities were seen in the basal ganglia and mesial temporal lobes on T1 or T2 scans. There was no abnormal contrast enhancement or structural abnormality noted. Serum sodium concentration was 120 mmol/L (reference range: 135–155 mmol/L). A cerebrospinal fluid (CSF) examination showed a normal opening pressure, with mild leukocytosis of 10 × 10^6^/L (reference range: < 5 × 10^6^/L), an elevated protein level of 1793.4 mg/L (reference range: 150-450 mg/L) and normal glucose. Oligoclonal band and the IgG index in CSF were within normal limits. Due to the involvement of the temporal lobes, herpes simplex virus (HSV) encephalitis was also considered in the beginning, but diagnostic HSV polymerase chain reaction (PCR) in the CSF was negative. An extensive serum and CSF evaluation for other viral, bacterial, and fungal etiologies was also negative. A chest scan revealed mild bilateral pulmonary infections, suggestive of aspiration pneumonia possibly due to seizures. Hematological tests and studies for screening malignancy, including an abdominal-CT scan, an ultrasound of the liver, gallbladder, spleen, pancreas and testicle, and serum tumor markers were unremarkable.

**Fig. 2:**
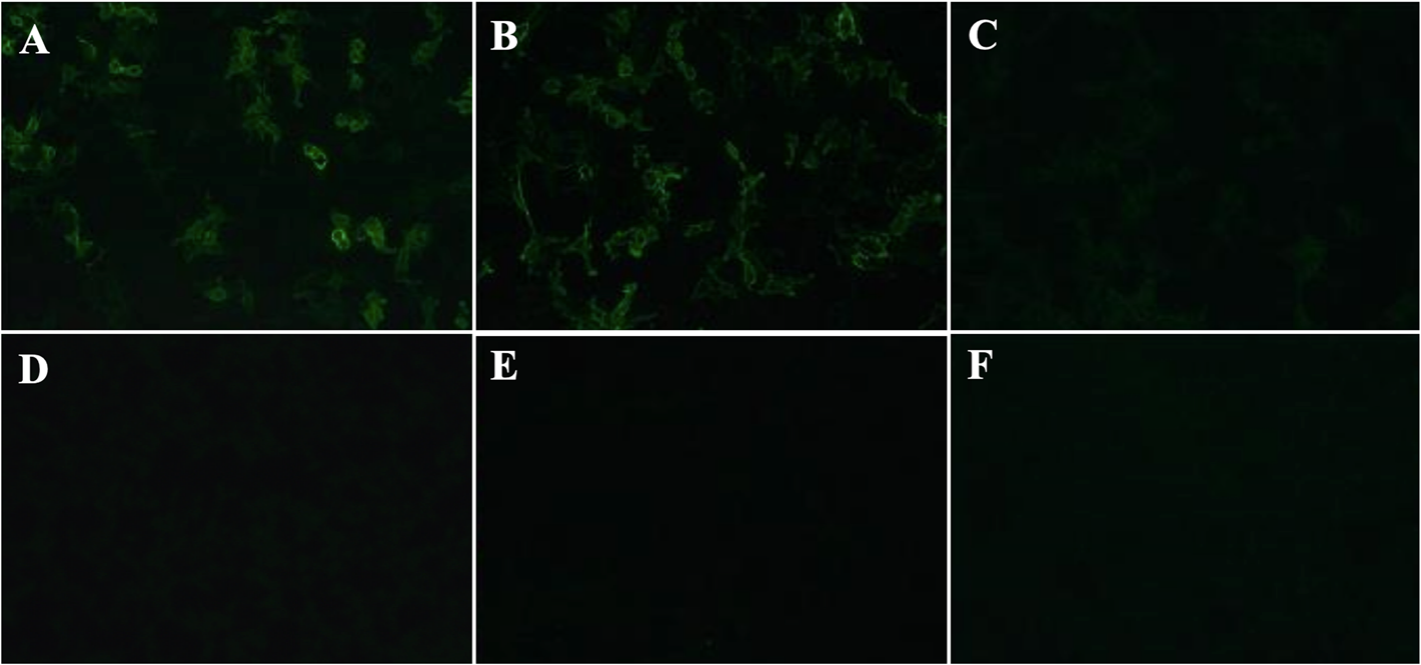
Anti-leucine-rich glioma-inactivated 1 (LGI1) antibodies in serum and CSF validated by cell-based assays. LGI1-IgG in CSF (**a**, 1:3.2) and serum (**b**, 1:32) were positive at disease onset, but negative in serum after 1 year (**c**). **d**-**f** were photos of control-transfected cells

**Fig. 3:**
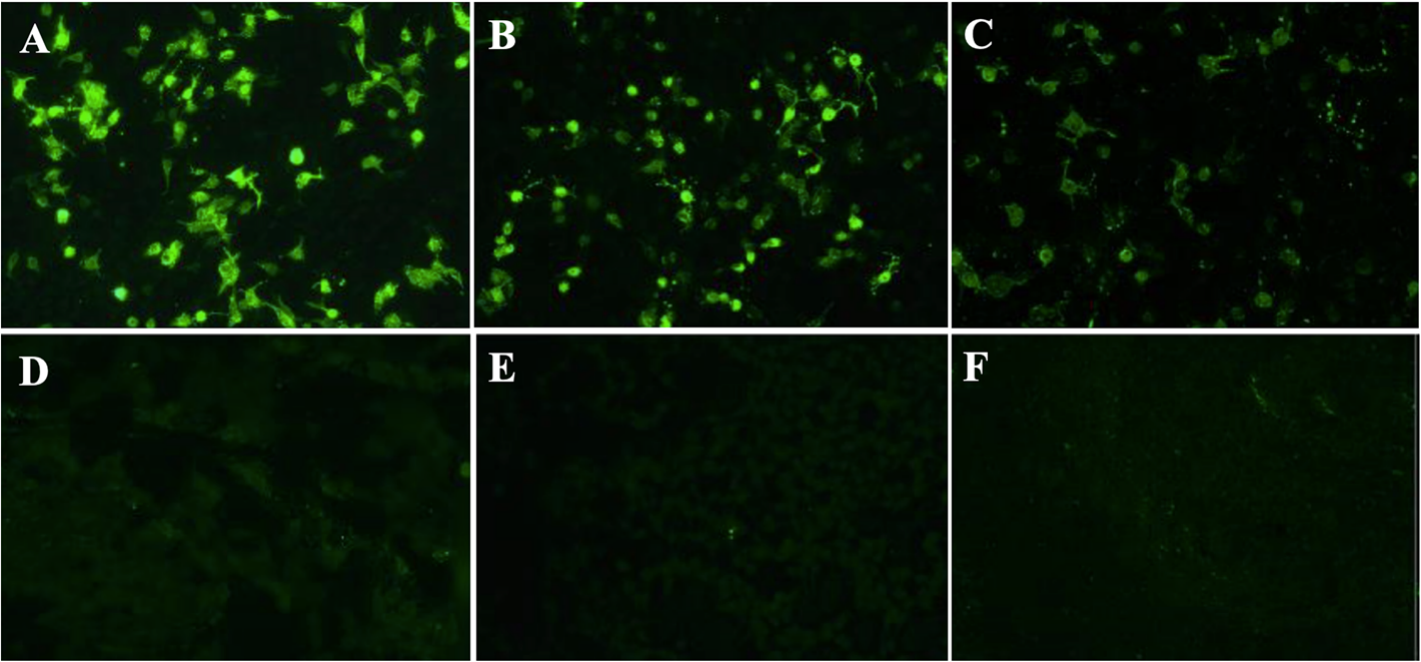
N-methyl-D-aspartate receptor (NMDAR) antibodies in serum and CSF validated by cell-based assays. NMDAR-IgG in CSF (**a**, 1:10) and serum (**b**, 1:100) were positive at disease onset, and remained positive in serum after 1 year (**c**, 1:32). **d**-**f** were photos of control-transfected cells

Cell-based assays (CBA) for clinical laboratory studies were conducted using Euroimmun IIFT kits: Autoimmune Encephalitis Mosaic 1 (FA 1121–1005-1), and/or NMDAR kits (FA112d-1005-51), according to the manufacturer’s instructions. The LGI1-IgG were positive both in CSF (1:3.2) and serum (1:32) (Fig. 2). In the meantime, we performed CBA tests for NMDAR-IgG, which also turned out positive in CSF (1:10) and serum (1:100) (Fig. 3). Other AE-related antibodies, such as anti-alpha-amino-3-hydroxy-5-methyl-4-isoxazolepropionic acid receptor 1 or 2, contactin-associated protein-like 2 receptor, dipeptidyl aminopeptidase-like protein 6 and anti-γ-aminobutyric acid-B receptor, were all negative in CSF and serum.

Given the concern for AE, the patient was treated with intravenous (IV) methylprednisolone (1 g/day for 3 days), which was then gradually tapered. Although HSV IgM and IgG antibodies were negative, IV acyclovir was empirically administered under the suspicion of HSE. Contemporarily he was treated with IV antibiotics, levetiracetam, and other supportive measures. There was significant improvement in his symptoms following treatment with IV methylprednisolone, including improved mentation, cessation of FBDS and alleviation of aggressive behaviors. The level of serum sodium gradually returned to normal. No adverse or unexpected events occurred during the treatment. He was discharged home on the 21st hospitalization day. Following discharge, he had an improvement in his mental status, with residual mild personality changes and short-term memory deficits. A long-term tapering schedule for oral prednisone was initiated prior to discharge. On follow-up assessment 1 year later, the patient remained seizure-free and had an improvement in his memory, although his daughter reported mild persistent personality changes. Repeated serum antibodies in January 2020 remained positive for NMDAR-IgG (1:32) (Fig. 2), but negative for LGI1-IgG (Fig. 3). Further cancer screening will be performed at future visits.

## Discussion and conclusions

We report the case of LGI1-LE with overlapping symptoms and simultaneous positive NMDAR antibodies. Our patient was an elderly man, who presented with subacute onset of recurrent GTCS followed by psychiatric symptoms, working memory deficits, sleep disorders, and FBDS. Based on the serum hyponatremia, CSF pleocytosis, bilateral brain abnormalities on FLAIR highly restricted to the medial temporal lobes, and positive LGI1-IgG in both CSF and serum, this case satisfies the recent criteria of definite LGI1-LE [3]. In view of typical disease course of LGI1-LE, initial symptoms often start with FBDS and focal seizures followed by cognitive decline. However, GTCS are relatively uncommon and usually manifest in the later stages of disease [5–8]. Intriguingly, the symptoms of our patient started with typical GTCS, and he displayed significant psychiatric symptoms early in the disease course, which are emerging as prominent clinical features of anti-NMDAR encephalitis [3]. In this regard, we performed the CBA tests for NMDAR-IgG, which turned out positive in both CSF and serum. To note, the serum NMDAR-IgG remained positive during follow-up, whereas the titer declined. The evidence of positive NMDAR-IgG in LGI1-LE raises concern for overlapping autoimmune complex.

LGl1 is a protein mainly expressed in the hippocampus and neocortex [2], that connects the presynaptic disintegrin and metalloproteinase domain-containing protein (ADAM) 23 to postsynaptic ADAM22, essential for inhibitory signal transmission from the presynaptic potassium channel to the post-synaptic AMPA receptors [9]. NMDARs are ligand-gated cation channels with crucial roles in synaptic transmission and plasticity. NMDARs are localized in neuronal post-synaptic membranes, predominantly found in the forebrain and limbic system, most notably the hippocampus. The pathogenesis of anti-NMDAR encephalitis may be attributed to antibody cross-linking and capping and internalization of the NMDARs, leading to decreased receptor density and the reduced synaptic functions of neurons [10, 11]. It is still not clear why both antibodies were present in our patient as their coexistence remains rare. The propensity towards overlapping disease versus isolated syndromes also needs further exploration. This may also help elucidate if either or both antibodies are pathogenic.

AE remains an under-recognized clinical syndrome but one where early and accurate detection is critical as prompt initiation of immunotherapy is closely associated with improved outcomes [12]. The treatment strategies and prognosis may be more complicated in patients with overlapping symptoms, and thus timely recognition of the potential concurrence of different antibodies is of great significance [4].

The highlight of this case is to provide the possibility of concurrence of NMDAR antibodies in LGI1-LE. The positive NMDAR-IgG may not be explained by previous HSE because the patient denied relative medical history and the diagnostic HSV PCR in the CSF was negative. However, there are indeed several limitations. First, we were not able to perform Immunohistochemistry to confirm CBA due to various restrictions. Nevertheless, since the CSF sample was evaluated twice by two independent observers and the serum NMDAR-IgG remained positive during follow-up, we presume the chance of a false-positive result of NMDAR antibody is relatively low. Second, the presence of serum autoantibodies may not necessarily suggest disease. Dysfunction of the NMDAR not associated with anti-NMDAR encephalitis has been implicated in schizophrenia, Alzheimer’s disease, and epilepsy [13]. However, given the evidence of dual positivity in both serum and CSF, and the declining of titers of the serum NMDAR-IgG during follow-up, we conclude that the NMDAR-IgG is probably of clinical relevance. Third, given that the observation has been made in a single patient, it is possible that it might be a circumstantial finding. Nevertheless, a high index of suspicion should be maintained to differentiate concomitant anti-NMDAR from presumed LGI1-LE, especially when the patients exhibit atypical and overlapping symptoms.

In summary, we highlight a patient of LGI1-LE with overlapping symptoms and simultaneous positive NMDAR antibodies. The current case provides the possibility of the concurrence of NMDAR antibodies in LGI1-LE, whereas the underlying mechanism remains elusive. It is necessary to evaluate the presence of NMDAR antibodies in certain LGI1-LE patients with unusual symptoms. Caution should be exercised in interpreting the observation, given the fact of a single-case study. Therefore, more studies are definitely needed to determine the exact relationship between the two antibodies.

## Data Availability

The datasets supporting the conclusions of this article are included within the article.

## Abbreviations

NMDAR: N-methyl-D-aspartate receptor
LGI1: Leucine-rich glioma-inactivated 1
GTCS: Generalized tonic–clonic seizures
LGI1-LE: Anti-leucine-rich glioma-inactivated 1 limbic encephalitis
FBDS: Facio-brachial dystonic seizures
AE: Autoimmune encephalitis
MRI: Magnetic resonance imaging
HSE: Herpes simplex virus encephalitis
FLAIR: Fluid attenuation inversion recovery
CSF: Cerebrospinal fluid
HSV: Herpes simplex virus
PCR: Polymerase chain reaction
CBA: Cell-based assays
IV: Intravenous
ADAM: Disintegrin and metalloproteinase domain-containing protein
